# A Brønsted Acid-Catalyzed Multicomponent Reaction for the Synthesis of Highly Functionalized γ-Lactam Derivatives

**DOI:** 10.3390/molecules24162951

**Published:** 2019-08-14

**Authors:** Xabier del Corte, Edorta Martinez de Marigorta, Francisco Palacios, Javier Vicario

**Affiliations:** Departamento de Química Orgánica I, Centro de Investigación y Estudios Avanzados “Lucio Lascaray”- Facultad de Farmacia, University of the Basque Country, UPV/EHU Paseo de la Universidad 7, 01006 Vitoria-Gasteiz, Spain

**Keywords:** γ-lactam, pyrrolidones, multicomponent reactions, organocatalysis

## Abstract

Brønsted acids catalyze a multicomponent reaction of benzaldehyde with amines and diethyl acetylenedicarboxylate to afford highly functionalized γ-lactam derivatives. The reaction consists of a Mannich reaction of an enamine to an imine, both generated in situ, promoted by a phosphoric acid catalyst and a subsequent intramolecular cyclization. The hydrolysis of the cyclic enamine substrate can provide enol derivatives and, moreover, a second attack of the amine on the carboxylate can afford amide derivatives. An optimization of the reaction conditions is presented in order to obtain selectively cyclic enamines that can afford the enol species after selective hydrolysis.

## 1. Introduction

**Figure 1:**
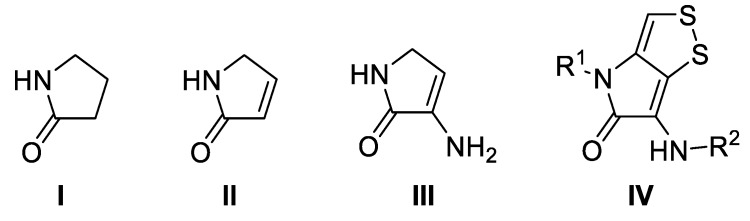
General structure of γ-lactams **I**, 1,5-dihydro-2*H*-pyrrol-2-ones **II**, 3-amino 1,5-dihydro-2*H*-pyrrol-2-ones **II**, and dithiopyrrolone antibiotics **IV**.

Multicomponent reactions (MCRs) [1,2] are valuable processes where three or more substrates, which are simultaneously (or almost) added, react in a single vessel to form a new structure that contains substantial portions of all the starting materials. Strecker, Hantzsch, Biginelli, Passerini, Gröbcke-Blackburn-Bienaymé, Kabachnik-Fields, or Ugi are some of the names of classical reactions that fit with this definition, and they are widely used in organic synthesis [1,2]. Due to the high degree of molecular diversity achieved in MCRs, they are now an essential tool in diversity-oriented synthesis [3,4], with huge potential in the field of medicinal chemistry [5,6]. Considering the relevance of the γ-lactam ring **I** (Figure 1) [7] and the increasing demand of potentially active compounds in medicinal sciences, MCR protocols were extensively used during the last decades for the synthesis of a wide number of densely functionalized γ-lactam derivatives [8,9]. In particular, 1,5-dihydro-2*H*-pyrrol-2-ones **II** (Figure 1) are conjugated unsaturated γ-lactam substrates with huge potential as intermediates in synthetic chemistry that also show assorted pharmacological activities [10,11,12,13].

Within this family of compounds, the structure of their 3-amino substituted derivatives **III** (Figure 1) contains the enamine moiety and, in addition to their obvious applications as synthetic intermediates in organic synthesis [14,15], their skeleton is also present in many new bioactive ingredients such as antimicrobials with anti-biofilm activity, caspase-3 inhibitors, antipyretics, or analgesics [16,17,18,19,20]. Moreover, these cyclic α-dehydro α,β-diamino acid derivatives contain the essential structure of dithiopyrrolone antibiotics **IV** (Figure 1) [21] and are key intermediates for the synthesis of Amaryllidaceae and *Sceletium* alkaloids [22,23].

**Scheme 1:**
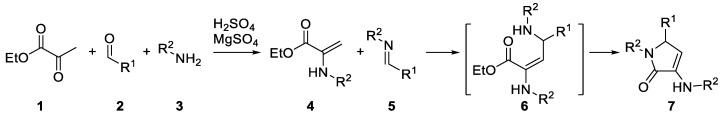
Three-component reaction of ethyl pyruvate **1**, aldehydes **2**, and amines **3**.

Several MCR procedures for the preparation of 3-amino 1,5-dihydro-2*H*-pyrrol-2-ones were reported to date [8]. In particular, some years ago, we reported a three-component reaction of ethyl pyruvate **1**, aldehydes **2**, and amines **3** mediated by sulfuric acid that yields very efficiently highly functionalized γ-lactam derivatives **7** [24]. In this reaction, an initial simultaneous condensation of amines **3** with both ethyl pyruvate **1** and aldehydes **2** leads to the formation of intermediate enamine **4** and aldimine **5** that undergo a subsequent Mannich reaction, followed by a cyclization reaction driven by the formation of an internal amide bond in the resulting adduct **6** (Scheme 1).

Based on this report, some authors later described several modifications of this synthetic procedure, and the uncatalyzed [25] or solvent-free [26] reaction, and the use of recyclable catalysts [27] were reported in the last few years. Interestingly, it was also established that such reaction can be performed under organocatalysis [28] and, taking the advantage of this fact, very recently, we achieved a highly enantioselective version of this reaction using 1,1′-bi-2-naphthol (BINOL)-derived chiral phosphoric acids as catalysts [29].

A similar multicomponent process, where dialkyl acetylenedicarboxylates are used instead of ethyl pyruvate, was also reported for the synthesis of 3-amino 1,5-dihydro-2*H*-pyrrol-2-ones. In this case, the nucleophilic addition of aromatic amines to the activated alkyne gives rise to a deactivated enamine intermediate and 0.5 equivalents of benzoic acid are required in order to promote the subsequent Mannich reaction [30]. Activation of this process was also described by the use of molecular iodine [31] or graphene-oxide nanosheets under solvent-free conditions [32]. In this context, organocatalysis is identified to be at the heart of greening of chemistry, because this branch of science is found to reduce the environmental impact of chemical processes. Therefore, in view of the demonstrated ability of phosphoric acids to catalyze the nucleophilic addition of pyruvate-derived enamines **4** to imines **5**, we thought that this activation could be extended to the enamines derived from dialkyl acetylenedicarboxylates. Consequently, continuing with the interest of our research group in the synthesis of nitrogenated heterocycles [33,34,35,36] and amino-acid derivatives [37,38,39,40], we report here the use of phosphoric acids as catalysts in a three-component reaction of amines, benzaldehyde, and diethyl acetylenedicarboxylate to afford densely functionalized γ-lactam derivatives.

## 2. Results

**Scheme 2:**
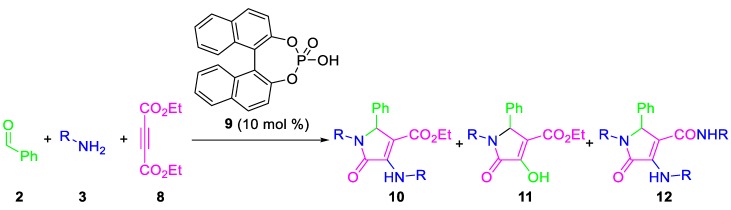
Three-component reaction of diethyl acetylenedicarboxilate **8**, benzaldehyde **2**, and amines **3**.

Based on our previous experience in MCRs for the synthesis of 3-amino 1,5-dihydro-2*H*-pyrrol-2-ones [11,14], we firstly used BINOL-derived phosphoric acid **9** as a Brønsted acid catalyst in the three-component reaction of benzaldehyde **2**, *p*-toluidine **3** (R = *p*-MeC_6_H_4_), and diethyl acetylenedicarboxylate **8** using refluxing dichloromethane as solvent (Scheme 2). However, only the enamine and/or imine intermediates that result from the reaction of amine substrate **3** with benzaldehyde **2** or alkyne **8** were observed in the crude (Table 1, Entry 1). Considering that our previously reported three-component reaction of ethyl pyruvate, benzaldehyde, and amines smoothly yields the corresponding 3-amino 1,5-dihydro-2*H*-pyrrol-2-ones, we thought that the increased steric hindrance, together with the additional deactivation present in the enamine intermediate when acetylenedicarboxylates **8** are used instead of pyruvate derivatives, may be the reason for the lack of reactivity in this case.

Then, we tried to perform the reaction at higher temperature and, although the same results were observed using tetrahydrofurane (THF) or dimethoxyethane (DME) as solvents (Table 1, Entries 2 and 3), the reaction in refluxing methyl *tert*-butylether (MTBE) proceeded in full conversion in a few hours, affording the expected 3-amino 1,5-dihydro-2*H*-pyrrol-2-one **10a** together with enol derivative **11a** [41], which may result from the hydrolysis of enamine moiety in **10a** (Table 1, Entry 4). The use of an excess of ethyl pyruvate in the parent MCR with benzaldehyde and amines proved to be very effective in reducing the reaction times and temperatures [29]; however, remarkably, when three equivalents of acetylene derivative **8** were used, no formation of γ-lactam derivatives **10a** or **11a** was observed due to the consumption of *p*-toluidine **3a** (R = *p*-MeC_6_H_4_) by reaction with the excess of diethyl acetylenedicarboxylate **8** (Table 1, Entry 5).

Better selectivity was observed when the reaction was performed using hot dioxane as solvent. In this case, amino 1,5-dihydro-2*H*-pyrrol-2-one **10a** was obtained together with a significant amount of amide derivative **12a**, which presumably results from the nucleophilic attack of amine on the ethyl carboxylate moiety in compound **10a** (Table 1, Entry 6). Finally, the selectivity of the reaction was further improved using toluene as the reaction solvent, and only a small amount (5%) of amide derivative **12a** was obtained together with γ-lactam **10a** (Table 1, Entry 7). Under the same conditions, the use of more nucleophilic *p*-anisidine **3b** (R = *p*-MeOC_6_H_4_) in the reaction yielded 1,5-dihydro-2*H*-pyrrol-2-one **10b** as the major product of the reaction although, in this case, together with a 30% of amide derivative **12b** (Table 1, Entry 8). In order to obtain exclusively amide substrate **12b**, four equivalents of amine were used under the same reaction conditions, but the same proportion of the products was observed (Table 1, Entry 9). However, the use of benzylamine **3c** (R = Bn) afforded exclusively 1,5-dihydro-2*H*-pyrrol-2-one **10c**, and no formation of enol **11c** or amide **12c** was observed (Table 1, Entry 10). The selectivity in this case could be explained by the lower steric crowding in the enamine moiety in benzylamine derivative **10c** if compared to the aromatic derivatives **10a** and **10b** [42].

**Scheme 3:**
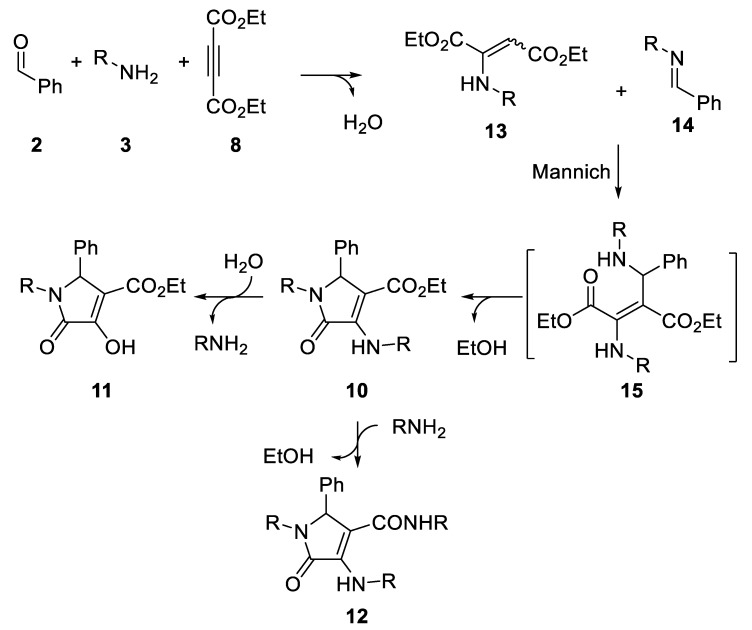
Reaction mechanism for the three-component reaction of diethyl acetylenedicarboxylate **8**, benzaldehyde **2**, and amines **3**.

In view of the three compounds observed, the reaction mechanism could start with an initial concomitant addition of amines **3** to acetylene carboxylate **8** and benzaldehyde **2** that affords enamine **13** and aldimine **14**. Both species **13** and **14** can be observed by ^1^H-NMR. Then, a subsequent Mannich reaction leads to the formation of adduct **15**, which undergoes an intramolecular cyclization by the formation of an internal amide bond between the amine and carboxylate moieties to afford enamine type γ-lactam **10**. Due to the presence of water and some remaining amine **3**, the γ-lactam **10** may afford enol type lactam **11** through hydrolysis of the enamine moiety or amide derivative **12**, through the displacement of ethanol by the amine (Scheme 3). This is supported by the fact that, using high-boiling-point solvents, no enol derivative **11** is observed, which may be due to the instantaneous evaporation of water at high reaction temperatures.

**Figure 2:**
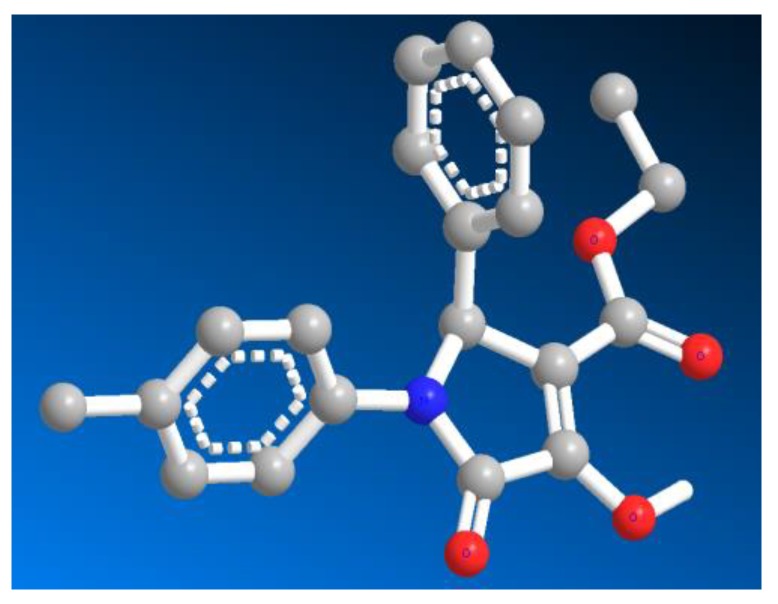
X-ray structure of **11a**. (blue ball, Nitrogen; gray ball, Carbon).

In our case, the three resulting γ-lactam structures **10**, **11**, and **12** could be separated in all the cases by simple chromatography, and they were fully characterized on the basis of their spectroscopic data. However, due to the structural resemblance between all the lactam derivatives, in order to unambiguously determine the identity of the substrates of the reaction, a single crystal of enol **11a** was prepared, and its X-ray diffraction structure was obtained (Figure 2). Key features of the crystal structure are the almost planar shape of the five-membered ring and the presence of a hydrogen bond between the enol hydrogen and the carboxylate group in a six-membered ring configuration rather than with the amide carboxylate, forming a five-membered ring.

**Scheme 4:**
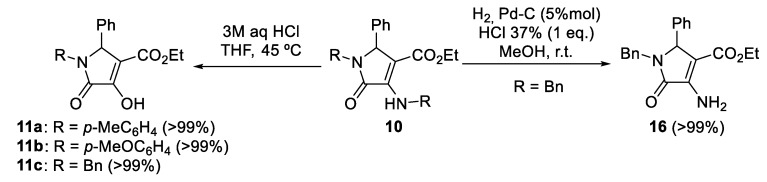
Preparation of enol derivatives **11** and deprotection of benzylamine derivative **10**.

In order to set up the optimal conditions for the preparation of enol derivatives **11**, we proposed the corresponding reactions starting from their parent 1,5-dihydro-2*H*-pyrrol-2-ones **10** (Scheme 4). Therefore, the hydrolysis of enamine moiety in **10** was performed by treatment of 1,5-dihydro-2*H*-pyrrol-2-ones **10** in the presence of aqueous hydrochloric acid in refluxing THF. Despite the strong acidic conditions, no trace of the products derived from the hydrolysis of ester of amide groups are observed and enol derivatives **11** are obtained in quantitative yields.

In addition, the treatment of benzylamine derivative γ-lactam **10** (R = Bn) with a catalytic amount of palladium under hydrogen atmosphere during several days led to the exclusive deprotection of the nitrogen at the enamine moiety in quantitative yield to afford lactam **16**. Remarkably, the benzyl group at the endocyclic nitrogen and the enamine double bond remained unaltered under those reaction conditions. Although the reaction times are very long, this process can be sped up by the addition of one equivalent of aqueous hydrochloric acid (Scheme 4).

**Figure 3:**
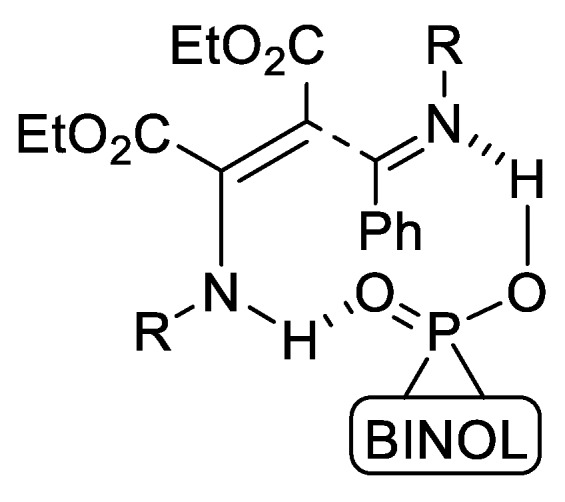
Transition state proposed for the Mannich reaction in the three-component reaction.

Taking into account the typical activation accepted by phosphoric acid catalysts [43,44,45], we propose a tentative transition state for the key Mannich reaction, where a dual activation of imine and enamine species takes place by the simultaneous formation of two hydrogen bonds between the phosphoryl oxygen and the acidic proton of the phosphoric acid group with the enamine proton and the iminic nitrogen, respectively (Figure 3).

According to the transition state proposed, we may expect substantial enantiomeric excesses for this reaction. However, when enantiomerically pure chiral phosphoric acids were used as catalysts, very poor enantioselectivities were observed with a maximum enantiomeric excess of 5%. This may be attributable to the high temperatures required for the reaction conditions because of the steric hindrance present in the enamine substrate, together with the additional deactivation of the nucleophile due to the presence of two carboxylate groups.

In conclusion, we report a Brønsted acid-catalyzed MCR procedure for the preparation of 3-amino 1,5-dihydro-2*H*-pyrrol-2-ones where diethyl acetylenedicarboxylate, amines, and benzaldehyde are used as substrates. This is the first example of such a reaction using phosphoric acids as catalyst. Moreover, we present nine highly functionalized γ-lactam derivatives, adding some molecular diversity to the already published substrates. The hydrolysis process of 1,5-dihydro-2*H*-pyrrol-2-ones from enamine substrates to the enol derivatives **11** was not previously reported.

## 3. Materials and Methods 

General. Solvents for extraction and chromatography were technical grade. All solvents used in reactions were freshly distilled from appropriate drying agents before use. All other reagents were recrystallized or distilled as necessary. All reactions were performed under an atmosphere of dry nitrogen. Analytical thin layer chromatography (TLC) was performed with silica gel 60 F_254_ plates. Visualization was accomplished by ultraviolet (UV) light. ^1^H-, ^13^C-, and ^31^P-NMR spectra were recorded on a Varian Unity Plus (at 300 MHz, 75 MHz, and 120 MHz, respectively, Advanced Research Facilities (SGIker), by the University of the Basque Country, Vitoria-Gasteiz, Spain) and on a Bruker Avance 400 (at 400 MHz, 100 MHz, and 160 MHz, respectively, Advanced Research Facilities (SGIker), by the University of the Basque Country, Vitoria-Gasteiz, Spain). Chemical shifts (δ) are reported in ppm relative to residual CHCl_3_ (δ = 7.26 ppm for ^1^H, and δ = 77.16 ppm for ^13^C-NMR). Coupling constants (*J*) are reported in Hertz. Data for ^1^H-NMR spectra are reported as follows: chemical shift, multiplicity, coupling constant, integration. Multiplicity abbreviations are as follows: s = singlet, d = doublet, t = triplet, q = quartet, m = multiplet, b = broad. ^13^C-NMR peak assignments were supported by distortionless enhanced polarization transfer (DEPT). High-resolution mass spectra (HRMS) were obtained by positive-ion electrospray ionization (ESI). Data are reported in the form *m*/*z* (intensity relative to base = 100). Infrared spectra (IR) were taken in a Nicolet iS10 Termo Scientific spectrometer as neat solids. Peaks are reported in cm^−1^. Copies of ^1^H- and ^13^C {^1^H} NMR spectra for γ-lactams **10**, **11**, **12**, and **16** are in Supplementary Materials.

Crystal structure determination for compound **11a**. Intensity data were collected on an Agilent Technologies Super-Nova diffractometer (Advanced Research Facilities (SGIker), by the University of the Basque Country, Leioa, Spain), which was equipped with monochromated Cu ka radiation (λ = 1.54184 Å) and Atlas CCD detector. Measurement was carried out at 150.00 (10) K with the help of an Oxford Cryostream 700 PLUS temperature device (Advanced Research Facilities (SGIker), by the University of the Basque Country, Leioa, Spain). Data frames were processed (united cell determination, analytical absorption correction with face indexing, intensity data integration, and correction for Lorentz and polarization effects) using the Crysalis software package (Version 1.171.37.31, release 14-01-2014 CryAlis171.NET, compiled Jan 14 2014, 18:38:05, Advanced Research Facilities (SGIker), by the University of the Basque Country, Leioa, Spain). The structure was solved using ShelXS (Sheldrick, 2008, Advanced Research Facilities (SGIker), by the University of the Basque Country, Leioa, Spain) [46] and refined by full-matrix least-squares with SHELXL-97 (Sheldrick, 2008, Advanced Research Facilities (SGIker), by the University of the Basque Country, Leioa, Spain) [47]. Final geometrical calculations were carried out with Mercury [48] and PLATON [49,50] as integrated in WinGX [51]. 

General procedure for the synthesis of 3-amino-1,5-dihydro-2*H*-pyrrol-2-ones **10**. A solution of benzaldehyde **2** (0.1 mL, 1 mmol), diethyl acetylenedicarboxylate **8** (0.16 mL, 1 mmol), amine **3** (2 mmol), phosphoric acid catalyst **9** (34.8 mg, 0.1 mmol), and anhydrous MgSO_4_ was stirred in toluene (5 mL) at 110 °C for 48 h. The volatiles were dried off at reduced pressure, and the crude residue was purified by column chromatography (AcOEt/hexanes) to afford pure lactams **10**. 

Ethyl 5-oxo-2-phenyl-1-(p-tolyl)-4-(p-tolylamino)-2,5-dihydro-1*H*-pyrrole-3-carboxylate (**10a**). The general procedure was followed, using *p*-toluidine (0.21 g, 2 mmol), affording 0.311 g (73%) of **10a** as a white solid. Melting point (m.p.) (Et_2_O) 154–155 °C. ^1^H-NMR (400 MHz, CDCl_3_): δ 8.17 (bs, 1H, NH), 7.34 (d, ^3^*J_HH_* = 8.5 Hz, 2H, 2× CHar), 7.26–7.21 (m, 5H, 5× CHar), 7.12 (d, ^3^*J_HH_* = 8.3 Hz, 2H, 2× CHar), 7.08 (d, *J* = 8.5 Hz, 2H, 2× CHar), 7.03 (d, ^3^*J_HH_* = 8.3 Hz, 2H, 2× CHar), 5.77 (s, 1H, CHN), 4.01 (q, ^3^*J_HH_* = 7.1 Hz, 2H, CH_2_ OEt), 2.33 (s, 3H, CH_3_), 2.23 (s, 3H, CH_3_), 1.01 (t, ^3^*J_HH_* = 7.1 Hz, 3H, CH_3_ OEt). ^13^C {^1^H} NMR (101 MHz, CDCl_3_) δ 164.7 (C=O ester), 164.1 (C=O amide), 142.7 (=C_quat_), 137.2 (C_quat_), 136.1 (C_quat_), 135.5 (C_quat_), 134.6 (C_quat_), 134.2 (C_quat_), 129.5 (2× CHar), 129.1 (2× CHar), 128.4 (2× CHar), 128.1 (CHar), 127.83 (2× CHar), 123.2 (2× CHar), 122.8 (2× CHar), 108.9 (=C_quat_), 63.3 (CHN), 60.2 (CH_2_ OEt), 21.1 (CH_3_), 21.0 (CH_3_), 14.0 (CH_3_ OEt). Fourier-transform IR (FTIR) (neat) ν_max_: 3289 (N–H), 1701 (C=O), 1679 (C=O), 1632 (C=C). HRMS (Q-TOF) *m*/*z* calculated for C_27_H_26_N_2_O_3_ [M]^+^ 426.1943, found 426.1950.

Ethyl 1-(4-methoxyphenyl)-4-((4-methoxyphenyl)amino)-5-oxo-2-phenyl-2,5-dihydro-1*H*-pyrrole-3-carboxylate (**10b**). The general procedure was followed, using *p*-anisidine (0.25 g, 2 mmol), affording 0.284 g (63%) of **10b** as a yellow solid. m.p. (Et_2_O) 116–117 °C. ^1^H-NMR (300 MHz, CDCl_3_) δ 8.20 (bs, 1H, NH), 7.29 (d, ^3^*J_HH_* = 9.1, 2H, 2× CHar), 7.24–7.18 (m, 5H, 5× CHar), 7.15 (d, ^3^*J_HH_* = 8.9 Hz, 2H, 2× CHar), 6.85 (d, ^3^*J_HH_* = 8.9 Hz, 2H, 2× CHar), 6.74 (d, ^3^*J_HH_* = 9.1, 2H, 2× CHar), 5.69 (bs, 1H, CHN), 4.01 (q, ^3^*J_HH_* = 7.1, 2H, CH_2_ OEt), 3.80 (s, 3H, CH_3_O), 3.71 (s, 3H, CH_3_O), 1.02 (t, ^3^*J_HH_* = 7.1, 3H, CH_3_ OEt). ^13^C {^1^H} NMR (75 MHz, CDCl_3_) δ 164.9 (C=O ester), 163.9 (C=O amide, 157.5 (C_quat_), 157.3 (C_quat_), 143.4 (=C_quat_), 137.3 (C_quat_), 131.6 (C_quat_), 129.7 (C_quat_), 128.4 (2× CHar), 128.1 (CHar), 127.9 (2× CHar), 125.1 (2× CHar), 124.8 (2× CHar), 114.1 (2× CHar), 113.8 (2× CHar), 107.9 (=C_quat_), 63.6 (CHN), 60.1 (CH_2_ OEt), 55.6 (CH_3_), 55.5(CH_3_), 14.1 (CH_3_ OEt). FTIR (neat) ν_max_: 3436 (N–H), 1704 (C=O), 1672 (C=O), 1629 (C=C). HRMS (Q-TOF) *m*/*z* calculated for C_22_H_15_Br_2_N_3_O_3_ [M]^+^ 458.1842, found 458.1844.

Ethyl 1-benzyl-4-(benzylamino)-5-oxo-2-phenyl-2,5-dihydro-1*H*-pyrrole-3-carboxylate (**10c**). The general procedure was followed, using benzylamine (0.21 g, 2 mmol), affording 0.234 g (58%) of **10c** as a white solid. m.p. (Et_2_O) 106–108 °C. ^1^H-NMR (400 MHz, DMSO-*d*_6_) δ 7.36 (m, 4H, 4× CHar), 7.34–7.21 (m, 8H, 7× Char + NH), 7.08 (m, 4H, 4× CHar), 5.09 (d, ^3^*J*_HH_ = 6.8 Hz, 2H, CH_2_ Bn), 4.95 (s, 1H, CHN), 4.86 (d, ^3^*J*_HH_ = 15.1 Hz, 1H, CH_2_ Bn), 3.96–3.81 (m, 2H, CH_2_ OEt), 3.65 (d, ^3^*J*_HH_ = 15.1 Hz, 1H, CH_2_ Bn), 0.91 (t, ^3^*J*_HH_ = 7.1 Hz, 3H, CH_3_ OEt). ^13^C {^1^H} NMR (101 MHz, DMSO-*d*_6_) δ 164.6 (C=O ester), 163.5 (C=O amide, 145.3 (=C_quat_), 139.8 (C_quat_), 137.0 (C_quat_), 136.2 (C_quat_), 128.0 (2× CHar), 127.9 (2× CHar), 127.8 (2× CHar), 127.5 (CHar), 127.3 (2× CHar), 127.1 (2× CHar), 126.8 (CHar), 126.7 (2× CHar), 126.4 (CHar), 103.4 (=C_quat_), 60.8 (CHN) , 58.4 (CH_2_ OEt), 45.3 (CH_2_ Bn), 43.4 (CH_2_ Bn), 13.3 (CH_3_ OEt). FTIR (neat) ν_max_: 3430 (N–H), 1691 (C=O), 1665 (C=O) 1624 (C=C). HRMS (Q-TOF) *m*/*z* calculated for C_22_H_15_F_2_N_3_O_3_ [M]^+^ 426.1943, found 426.1942.

General procedure for the hydrolysis of compounds **10**. To 10 mL of a 3 M HCl/THF (1:1) solution, compound **10** (0.5 mmol) was added; the mixture was heated to 75 °C and stirred overnight. The reaction was monitored by TLC and, once it was finished, the mixture was concentrated under reduced pressure to eliminate the THF, washed with 3 M NaOH (2× 5 mL) and H_2_O (2× 5mL), and extracted with ethyl acetate. The combined organic phases were dried with anhydrous Mg_2_SO_4_, and the crude residue was crystalized in Et_2_O: pentane.

Ethyl 4-hydroxy-5-oxo-2-phenyl-1-(p-tolyl)-2,5-dihydro-1*H*-pyrrole-3-carboxylate (**11a**). The general procedure was followed, affording 0.161 g (95%) of **11a** as a white solid. m.p. (Et_2_O) 170–172 °C. ^1^H-NMR (300 MHz, CDCl_3_) δ 9.19 (bs, 1H, OH), 7.38 (d, ^3^*J_HH_* = 8.2 Hz, 2H, 2× CHar), 7.32–7.25 (m, 5H, 5× CHar), 7.09 (d, ^3^*J_HH_* = 8.2 Hz, 2H, 2× CHar), 5.74 (s, 1H, CHN), 4.20 (q, ^3^*J_HH_* =, 7.1 Hz, 2H, CH_2_ OEt) 2.26 (s, 3H), 1.20 (t, ^3^*J_HH_* = 7.1Hz, 3H, CH_3_ OEt). ^13^C {^1^H} NMR (75 MHz, CDCl_3_) δ 165.0 (C=O ester), 162.9 (C=O amide), 156.4 (=C_quat_), 135.7 (C_quat_), 135.3 (C_quat_), 133.7 (C_quat_), 129.6 (2× CHar), 128.6 (2× CHar), 128.5 (CHar), 127.6 (2× CHar), 122.4 (2× CHar), 113.1 (=C_quat_), 61.8 (CHN), 61.2 (CH_2_ OEt), 20.9 (CH_3_), 14.0 (CH_3_ OEt). FTIR (neat) ν_max_: 3425 (O–H), 1704 (C=O), 1675 (C=O), 1643 (C=C). HRMS (Q-TOF) *m*/*z* calculated for C_27_H_26_N_2_O_3_ [M]^+^ 337.1314, found 337.1319.

Ethyl 4-hydroxy-1-(4-methoxyphenyl)-5-oxo-2-phenyl-2,5-dihydro-1*H*-pyrrole-3-carboxylate (**11b**). The general procedure was followed, affording 0.162 g (92%) of **11b** as a white solid. m.p. (Et_2_O) 182 °C (dec.). ^1^H-NMR (300 MHz, CDCl_3_) δ 9.05 (bs, 1H, OH), 7.30 (d, ^3^*J_HH_* = 8.9 Hz, 2H, 2× CHar), 7.24–7.16 (m, 5H, 5× CHar), 6.79 (d, ^3^*J_HH_* = 8.8 Hz, 2H, 2× CHar), 5.63 (s, 1H, CHN), 4.17 (q, ^3^*J_HH_* = 7.1 Hz, 2H, CH_2_ OEt), 3.72 (s, 3H, CH_3_), 1.16 (t, ^3^*J_HH_* = 7.1 Hz, 3H, CH_2_ OEt). ^13^C {^1^H} NMR δ 165.4 (C=O ester), 162.8 (C=O amide), 157.7 (C_quat_), 157.1 (=C_quat_), 135.3 (C_quat_), 129.3 (C_quat_), 128.7 (2× CHar), 128.6 (2× CHar), 127.7 (2× CHar), 124.5 (2× CHar), 120.5 (CHar), 114.4 (2× CHar), 113.0 (=C_quat_), 62.2 (CHN), 61.3 (CH_2_ OEt), 55.5 (CH_3_), 14.1 (CH_3_ OEt). FTIR (neat) ν_max_: 3431 (O–H), 1711 (C=O), 1677 (C=O), 1653 (C=CH). HRMS (Q-TOF) *m*/*z* calculated for C_27_H_26_N_2_O_3_ [M]^+^ 353.1263, found 353.1268.

Ethyl 1-benzyl-4-hydroxy-5-oxo-2-phenyl-2,5-dihydro-1*H*-pyrrole-3-carboxylate (**11c**). The general procedure was followed, affording 0.157 g (94%) of **11c** as a white solid. m.p. (Et_2_O) 178–179 °C. ^1^H-NMR (300 MHz, CDCl_3_) δ 9.11 (bs, 1H, OH), 7.39–7.33 (m, 3H, 3× CHar), 7.32–7.27 (m, 3H, 3× CHar), 7.15–7.08 (m, 4H, 4× CHar), 5.20 (d, ^3^*J_HH_* = 14.8 Hz, 1H, CH_2_ Bn), 4.88 (s, 1H, CHN), 4.08 (q, ^3^*J_HH_* = 7.2, 2H, , CH_2_ OEt), 3.55 (d, ^3^*J_HH_* = 14.8 Hz, 1H, CH_2_ Bn), 1.06 (t, ^3^*J_HH_* = 7.1 Hz, 3H, CH_3_ OEt). ^13^C {^1^H} NMR (75 MHz, CDCl_3_) δ 165.59 (C_quat_), 163.59 (C_quat_), 157.91 (C_quat_), 136.43 (C_quat_), 134.68 (C_quat_), 128.97 (CH), 128.68 (CH), 128.02 (CH), 127.98 (CH), 113.37 (C_quat_), 61.14 (CH_2_), 59.75 (CH), 44.11 (CH_2_), 13.97 (CH_3_). FTIR (neat) ν_max_: 3450 (N–H), 1735 (C=O), 1675 (C=O), 1632 (C=C). HRMS (Q-TOF) *m*/*z* calculated for C_27_H_26_N_2_O_3_ [M]^+^ 337.1314, found 337.1333.

General procedure for the isolation of amides **12.** A solution of benzaldehyde **2** (0.1 mL, 1 mmol), diethyl acetylenedicarboxylate **8** (0.16 mL, 1 mmol), amine **3** (2 mmol), phosphoric acid catalyst **9** (34.8 mg, 0.1 mmol), and anhydrous MgSO_4_ was stirred in toluene (5 mL) at 110 °C for 48 h. The volatiles were dried off at reduced pressure, and the crude residue was purified by column chromatography (AcOEt/hexanes) to afford pure lactams **12**.

5-oxo-2-phenyl-N,1-di-p-tolyl-4-(p-tolylamino)-2,5-dihydro-1*H*-pyrrole-3-carboxamide (**12a**). The general procedure was followed, affording 0.02 g (4%) of **12a** as a white solid. m.p. (Et_2_O) 226 °C (dec.). (300 MHz, CDCl_3_) δ 8.31 (bs, 1H, NH), 7.38–7.28 (m, 6H, 6× CHar), 7.11–7.04 (m, 7H, 7× CHar), 6.96 (d, ^3^*J_HH_* = 8.5 Hz, 2H, 2× CHar), 6.84 (d, ^3^*J_HH_* = 8.5 Hz, 2H, 2× CHar), 6.63 (bs, 1H, NH), 5.85 (s, 1H, CHN), 2.28 (s, 3H,CH_3_), 2.25 (s, 3H, CH_3_), 2.24 (s, 3H, CH_3_). ^13^C {^1^H} NMR (75 MHz, CDCl_3_) (75 MHz, CDCl_3_) δ 164.75 (C=O), 162.12 (C=O), 139.1 (=C_quat_), 136.6 (C_quat_), 136.1 (C_quat_), 135.8 (C_quat_), 134.8 (C_quat_), 134.6 (C_quat_), 133.9 (C_quat_), 133.8 (C_quat_), 129.7 (4× CHar), 129.5 (2× CHar), 129.4 (2× CHar), 129.3 (CHar), 128.0 (2× CHar), 123.3 (2× CHar), 122.5 (2× CHar), 119.8 (2× CHar), 112.4 (=C_quat_), 63.8 (CHN), 21.1 (CH_3_), 21.0 (CH_3_), 21.0 (CH_3_). FTIR (neat) ν_max_: 3309 (N–H), 3251 (N–H), 1685 (C=O), 1632 (C=C). HRMS (Q-TOF) *m*/*z* calculated for C_27_H_26_N_2_O_3_ [M]^+^ 487.22598, found 487.2255.

*N*,1-bis(4-methoxyphenyl)-4-((4-methoxyphenyl)amino)-5-oxo-2-phenyl-2,5-dihydro-1H-pyrrole-3-carboxamide (**12b**). The general procedure was followed, affording 0.07 g (13%) of **12b** as a white solid. m.p. (Et_2_O) 228–229 °C. ^1^H-NMR (400 MHz, CDCl_3_) δ 8.46 (bs, 1H, NH), 7.37–7.28 (m, 5H, 5× CHar), 7.26–7.22 (m, 2H, 2× CHar), 7.17 (d, ^3^*J_HH_* = 8.8 Hz, 2H, 2× CHar), 6.90 (d, ^3^*J_HH_* = 9.1 Hz, 2H, 2× CHar), 6.82 (d, ^3^*J_HH_* = 8.9 Hz, 2H, 2× CHar), 6.78 (d, ^3^*J_HH_* = 9.1 Hz, 2H, 2× CHar), 6.71 (d, ^3^*J_HH_* = 9.1 Hz, 2H, 2× CHar), 6.56 (bs, 1H, NH), 5.76 (s, 1H, CHN), 3.74 (s, 3H, CH_3_), 3.73 (s, 3H, CH_3_), 3.72 (s, 3H, CH_3_). ^13^C {^1^H} NMR (101 MHz, CDCl_3_) δ 164.6 (C=O), 162.4 (C=O), 157.8 (C_quat_), 157.2 (C_quat_), 156.5 (C_quat_), 140.2 (=C_quat_), 136.7 (C_quat_), 131.6 (C_quat_), 130.5 (C_quat_), 129.5 (2× CHar), 129.3 (CHar), 128.0 (2× CHar), 125.4 (2× CHar), 124.5 (2× CHar), 121.5 (2× CHar), 114.3 (2× CHar), 114.3 (2× CHar), 114.1 (2× CHar), 110.9 (=C_quat_), 64.1 (CHN), 55.6 (CH_3_), 55.6 (CH_3_), 55.5 (CH_3_). FTIR (neat) ν_max_: 3344 (N–H), 3286 (N–H), 1662 (C=O), 1682 (C=O), 1632 (C=C). HRMS (Q-TOF) *m*/*z* calculated for C_27_H_26_N_2_O_3_ [M]^+^ 535.2107, found 535.2105.

Ethyl 4-amino-1-benzyl-5-oxo-2-phenyl-2,5-dihydro-1*H*-pyrrole-3-carboxylate (**16**). A mixture of **10c** (21.3 mg, 0.5 mmol), 10% palladium on carbon (276 mg, 0.025 mmol), and 37% HCl (0.05 mL, 0.5 mmol) in methanol (30 mL) was stirred for 10 h under hydrogen pressure at 70 psi. The reaction mixture was filtered through Celite, and the filtered solution was treated with NaHCO_3_ until neutral and extracted with dichloromethane (3 × 15 mL). The combined organic fractions were dried with anhydrous MgSO_4_, and distilled off at reduced pressure; the residue was crystallized in Et_2_O/pentane (1:2) to afford 0.163 g (97%) of **11c** as a white solid. m.p. (Et_2_O) 139–142 °C. ^1^H-NMR (400 MHz, CDCl_3_) δ 7.37–7.27 (m, 6H, 6× CHar), 7.18–7.05 (m, 4H, 4× CHar), 5.74 (bs, 2H, NH), 5.13 (d, ^3^*J_HH_* = 14.8 Hz, 1H, CH_2_ Bn), 4.89 (s, 1H, CHN), 4.10–3.87 (m, 2H, CH_2_ OEt), 3.57 (d, ^3^*J_HH_* = 14.8 Hz, 1H, CH_2_ Bn), 1.05 (t, ^3^*J_HH_* = 7.1 Hz, 3H, CH_3_ OEt). ^13^C {^1^H} NMR (75 MHz, CDCl_3_) δ 165.5 (C=O ester), 165.0 (C=O amide), 145.9 (=C_quat_), 136.6 (C_quat_), 136.6 (C_quat_), 128.9 (2× CHar), 128.7 (2× CHar), 128.5 (2× CHar), 128.4 (CHar), 128.0 (2× CHar), 127.9 (CHar), 104.8 (=C_quat_), 61.5 (CHN) , 59.8 (CH_2_ OEt), 44.2 (CH_2_ Bn), 14.2 (CH_3_ OEt). FTIR (neat) ν_max_: 3450 and 3319 (N–H_2_), 1685 (C=O), 1654 (C=O), 1643 (C=C). HRMS (Q-TOF) *m*/*z* calculated for C_27_H_26_N_2_O_3_ [M]^+^ 336.1474, found 336.1476.

## Figures and Tables

**Table 1:** Three-component reaction of ethyl pyruvate **1**, benzaldehyde **2**, and amines **3**.

Entry	R	2/3/8	Solvent	T (°C)	Yield (%) ^1^	10/11/12 ^2^
1	*p*-MeC_6_H_4_	1/2/1	CH_2_Cl_2_	40	0	n.d.
2	*p*-MeC_6_H_4_	1/2/1	THF	65	0	n.d.
3	*p*-MeC_6_H_4_	1/2/1	DME	85	0	n.d.
4	*p*-MeC_6_H_4_	1/2/1	MTBE	55	72	40/60/0
5	*p*-MeC_6_H_4_	1/2/3	MTBE	55	0	n.d.
6	*p*-MeC_6_H_4_	1/2/1	Dioxane	101	81	80/0/20
7	*p*-MeC_6_H_4_	1/2/1	Toluene	110	77	95/0/5
8	*p*-MeOC_6_H_4_	1/2/1	Toluene	110	76	70/0/30
9	*p*-MeOC_6_H_4_	1/4/1	Toluene	110	76	70/0/30
10	Bn	1/2/1	Toluene	110	58	100/0/0

^1^ Isolated total yield. ^2^ Determined by ^1^H-NMR. n.d.—not determined.

## Supplementary Materials

Copies of ^1^H- and ^13^C {^1^H} NMR spectra for γ-lactams **10**, **11**, **12**, and **16** are available online. CCDC 1938640 contains the supplementary crystallographic data for this paper (compound **11a**). The data can be obtained free of charge from the Cambridge Crystallographic Data Center via www.ccdc.cam.ac.uk/structures
