# Supplementary material for: A Brønsted Acid-Catalyzed Multicomponent Reaction for the Synthesis of Highly Functionalized γ-Lactam Derivatives
Source: Molecules. 2019 Aug 14;24(16):2951. doi: 10.3390/molecules24162951 (PMC6719937; doi:10.3390/molecules24162951)
Supplement: Supplementary file 1 [file molecules-24-02951-s001.pdf]

Supporting Information

Brønsted acid-catalyzed multicomponent reaction for the synthesis of highly functionalized γ-lactam derivatives.

Xabier del Corte [1], Edorta Martinez de Marigorta [1], Francisco Palacios [1,*] and Javier Vicario [1,*]

* francisco.palacios@ehu.eus / javier.vicario@ehu.eus Departamento de Química Orgánica I, Centro de Investigación y Estudios Avanzados "Lucio Lascaray"- Facultad de Farmacia, University of the Basque Country, UPV/EHU Paseo de la Universidad 7, 01006 Vitoria-Gasteiz, SPAIN

Table of contents

1. ^{1}H NMR and ^{13}C NMR spectra of compounds **10**, **11**, **12** and **16** **S2-S10**
2. Crystal structure determination for compound **11a**. **S11**

1. ^{1}H and ^{13}C NMR spectra of compounds 10, 11, 12 and 16.

Ethyl 5-oxo-2-phenyl-1-(p-tolyl)-4-(p-tolylamino)-2,5-dihydro-1H-pyrrole-3-carboxylate. (10a).
^{1}H NMR (400 MHz, $CDCl_3$)

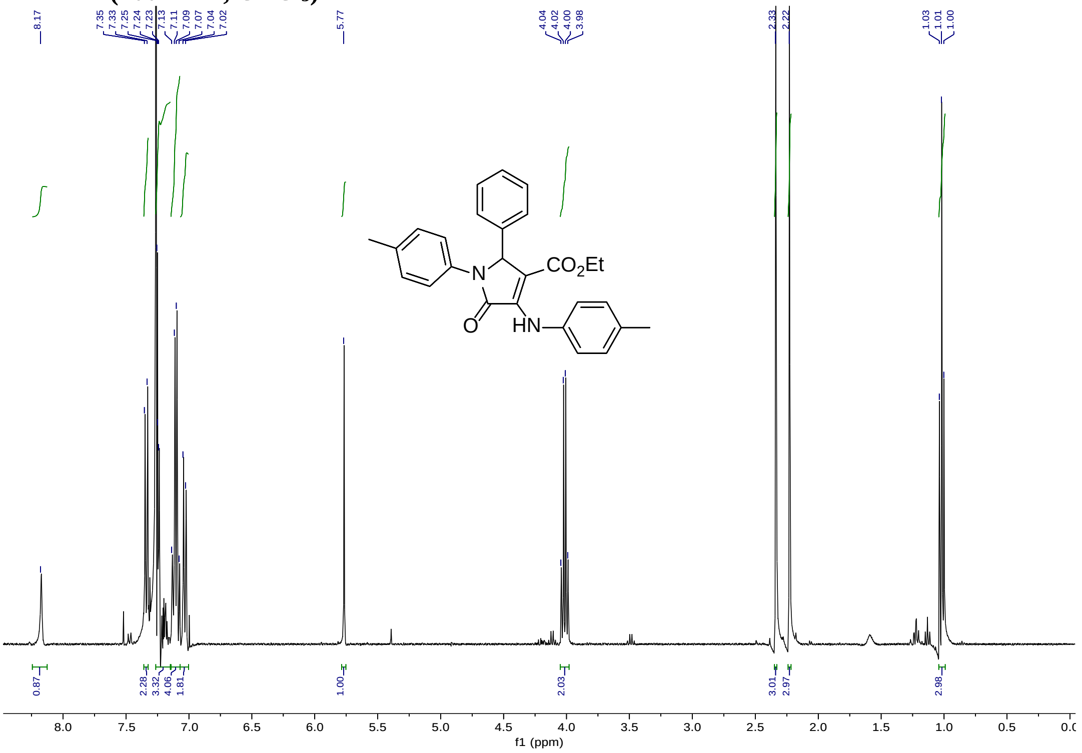

^{13}C NMR (101 MHz, $CDCl_3$)

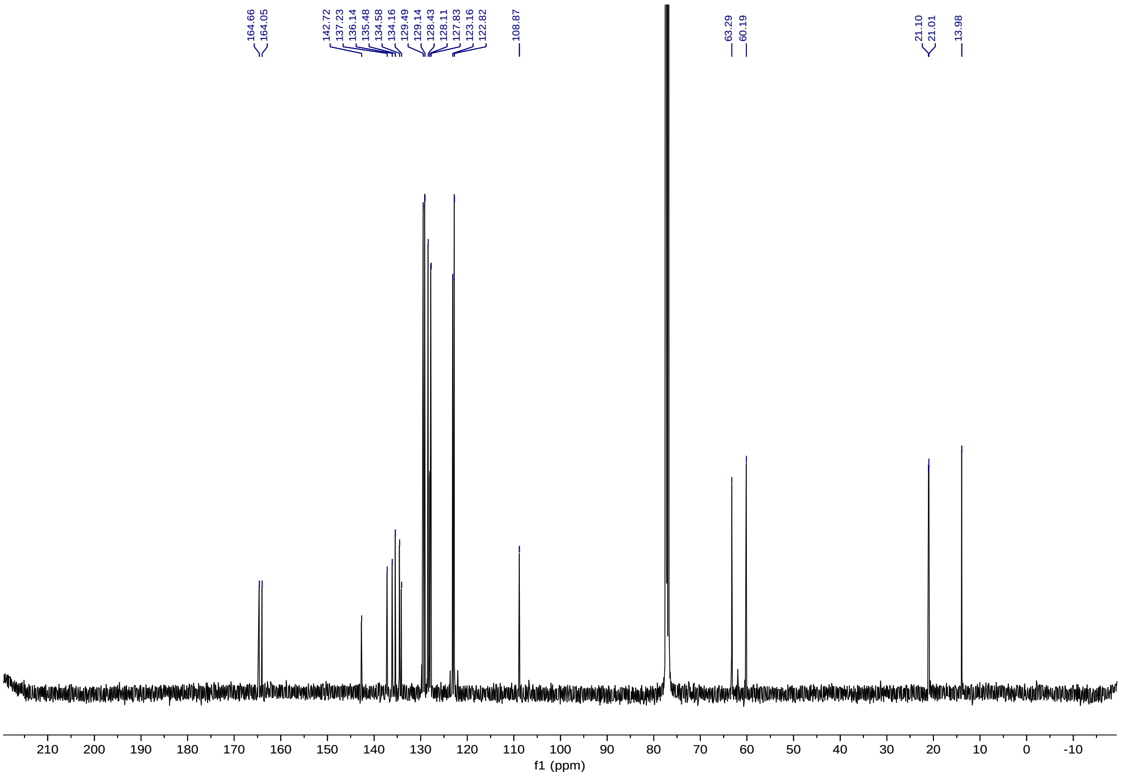

Ethyl 1-(4-methoxyphenyl)-4-((4-methoxyphenyl)amino)-5-oxo-2-phenyl-2,5-dihydro-1H-pyrrole-3-carboxylate (10b).

^{1}H NMR (300 MHz, $CDCl_3$)

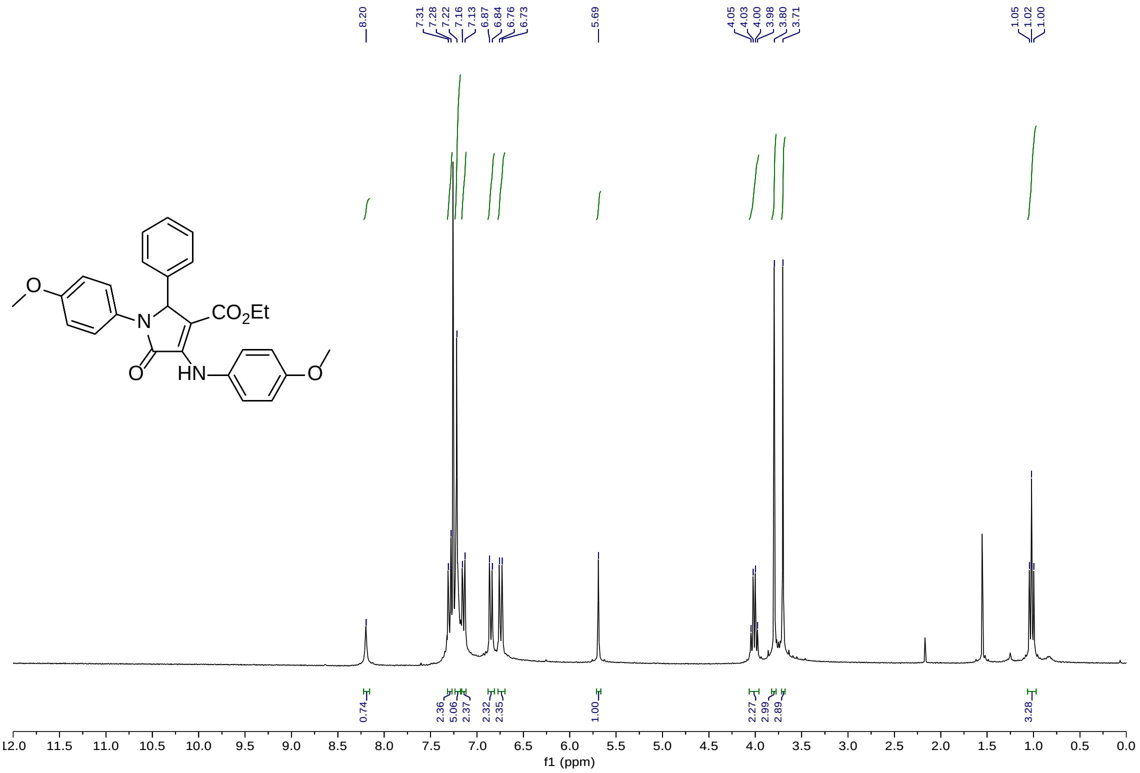

^{13}C NMR (75 MHz, $CDCl_3$)

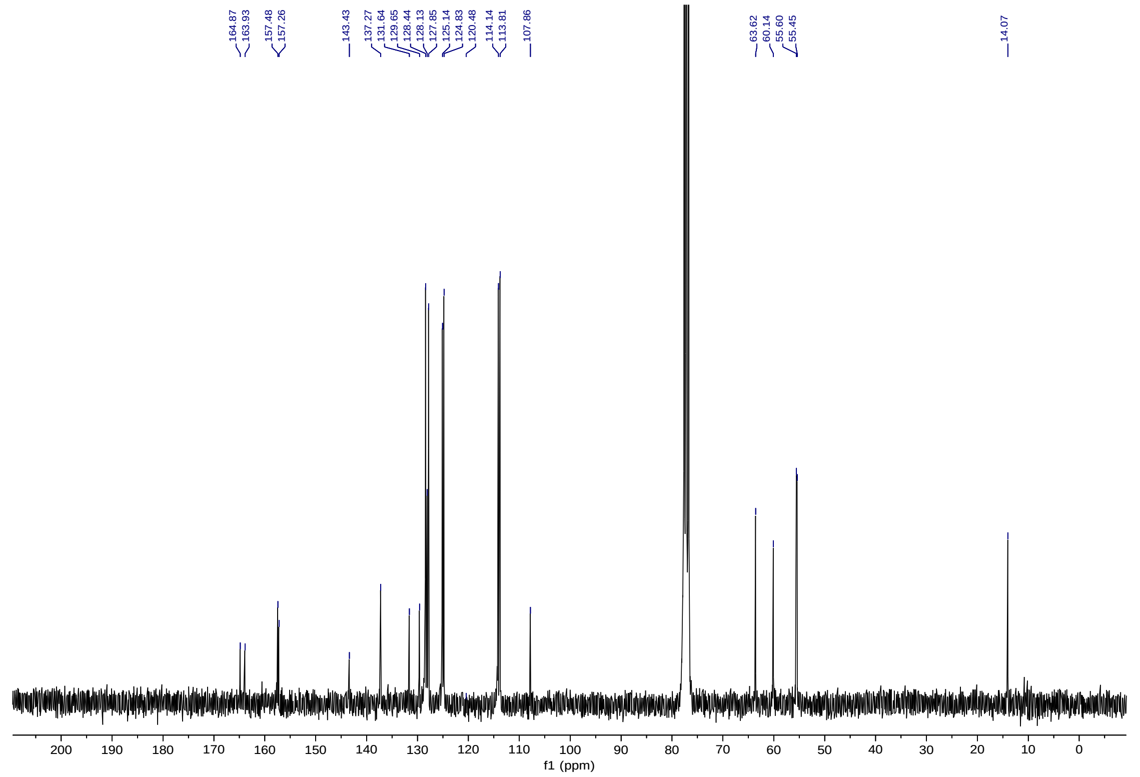

Ethyl 1-benzyl-4-(benzylamino)-5-oxo-2-phenyl-2,5-dihydro-1H-pyrrole-3-carboxylate (10c).

^{1}H NMR (400 MHz, DMSO-*d*$_6$)

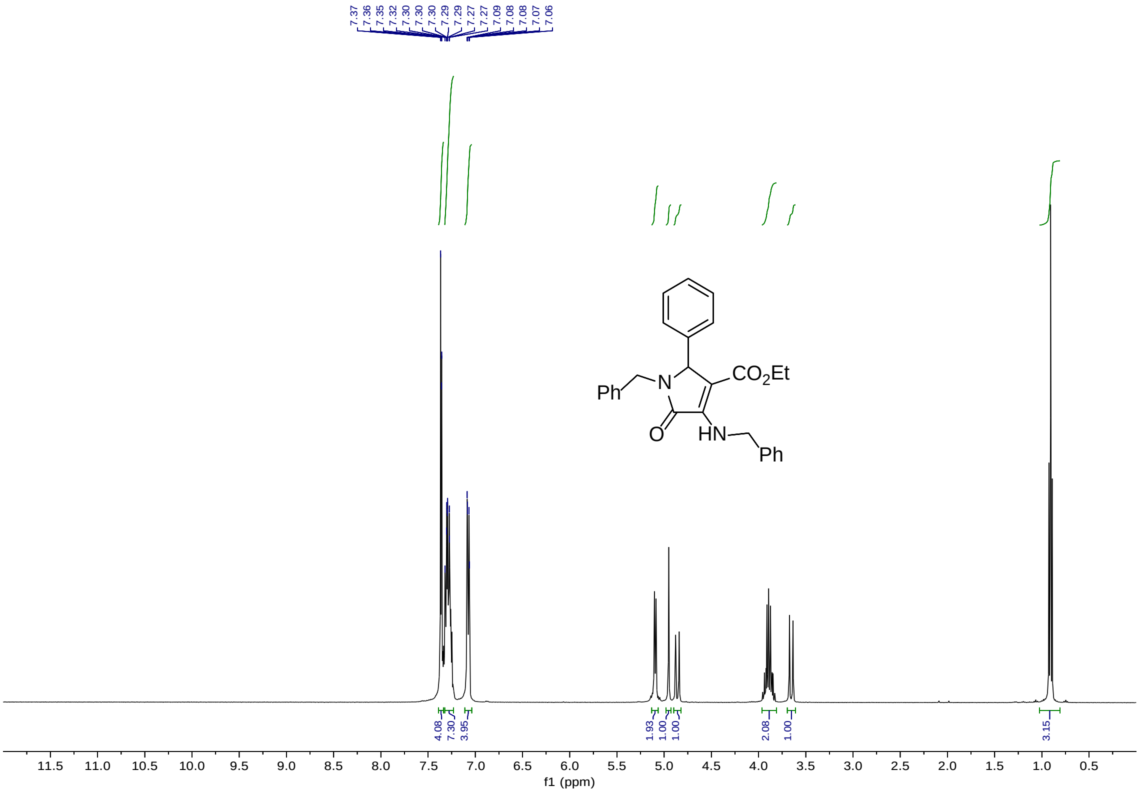

^{13}C NMR (101 MHz, DMSO *d*$_6$)

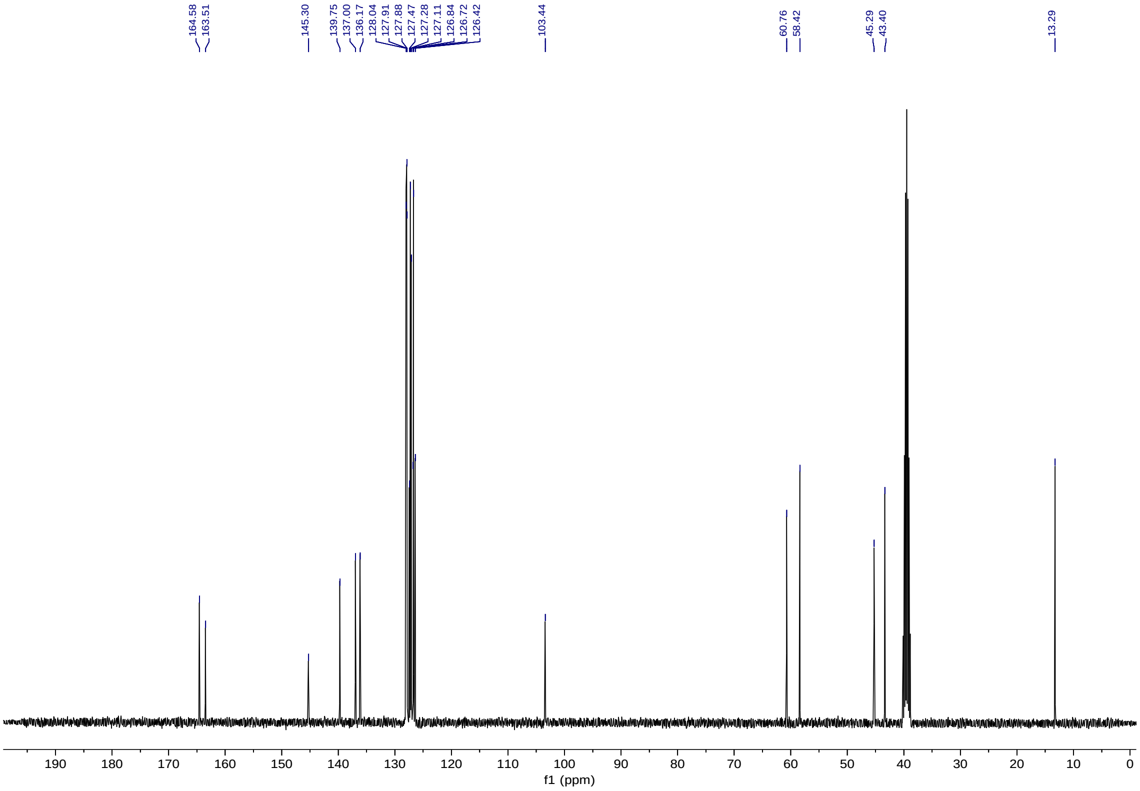

Ethyl 4-hydroxy-5-oxo-2-phenyl-1-(p-tolyl)-2,5-dihydro-1H-pyrrole-3-carboxylate. (11a).

^{1}H NMR (300 MHz, CDCl3)

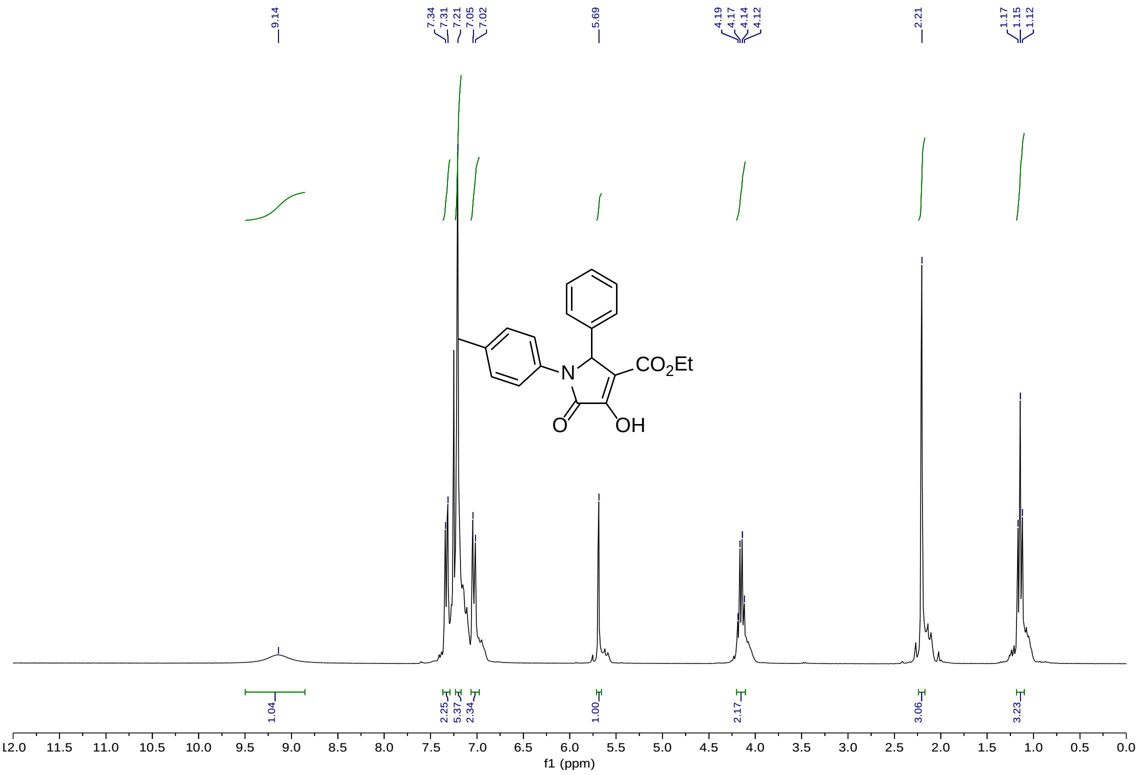

^{13}C NMR (75 MHz, CDCl3

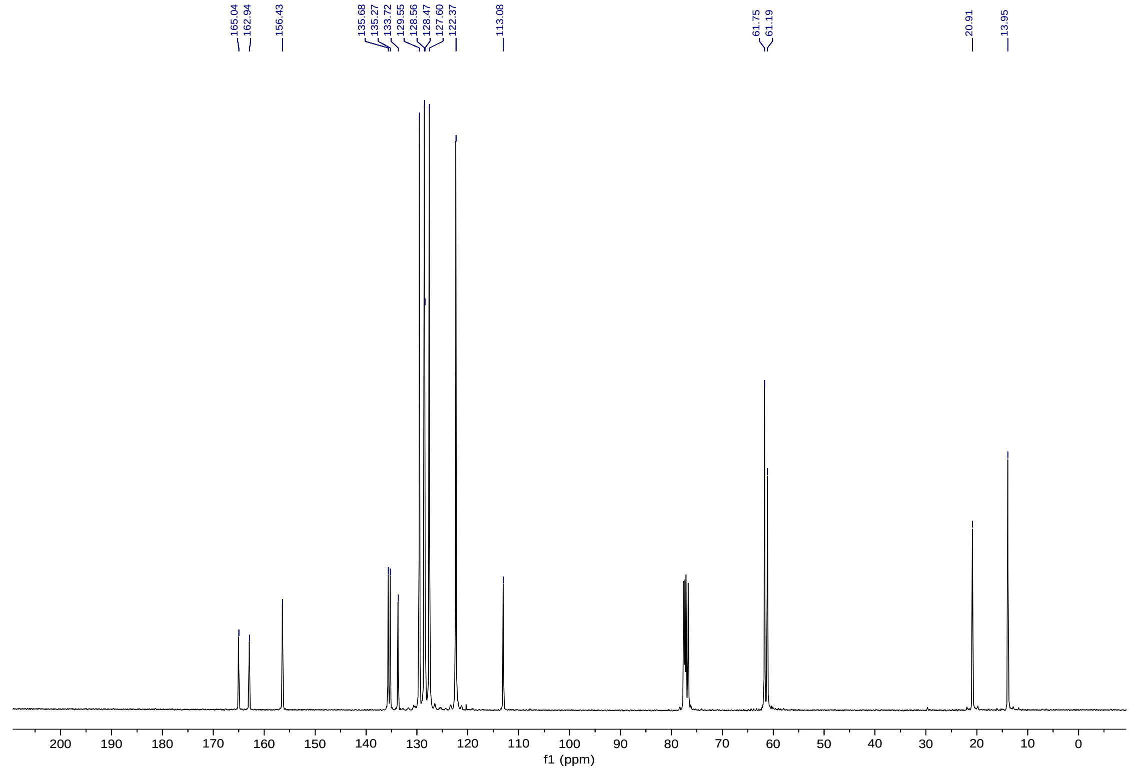

Ethyl 4-hydroxy-1-(4-methoxyphenyl)-5-oxo-2-phenyl-2,5-dihydro-1H-pyrrole-3-carboxylate. (11b).

^{1}H NMR (300 MHz, $CDCl_3$)

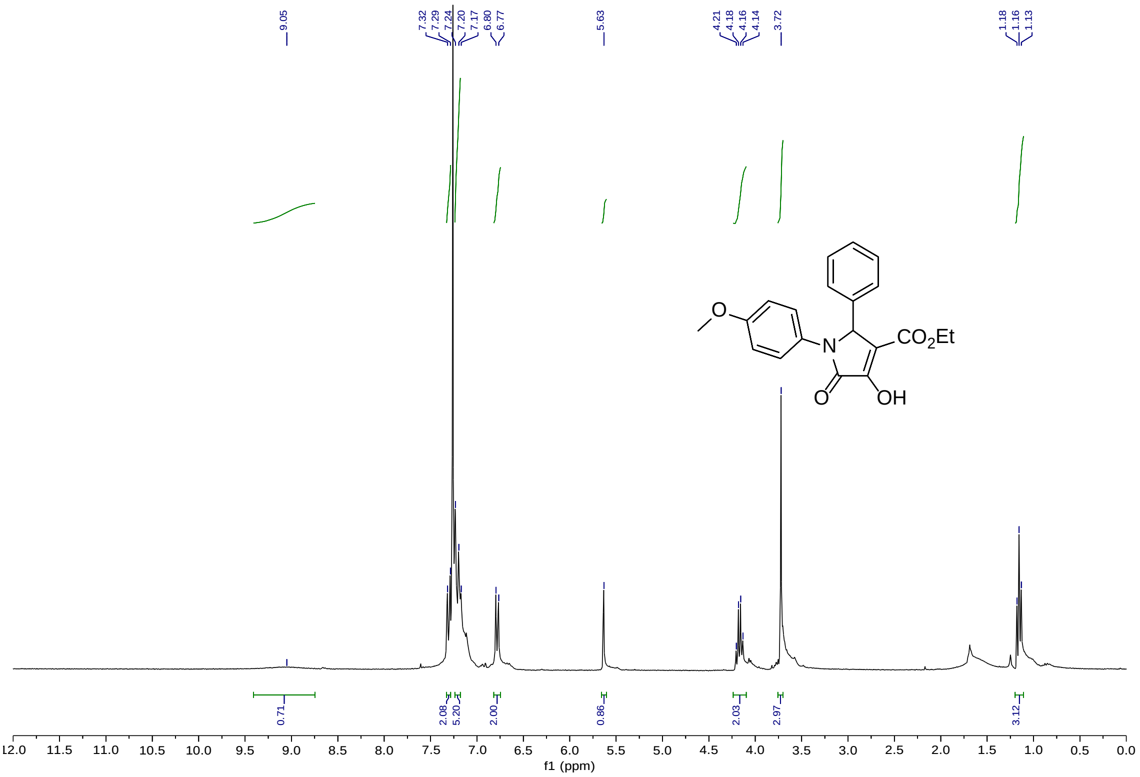

^{13}C NMR (75 MHz, $CDCl_3$)

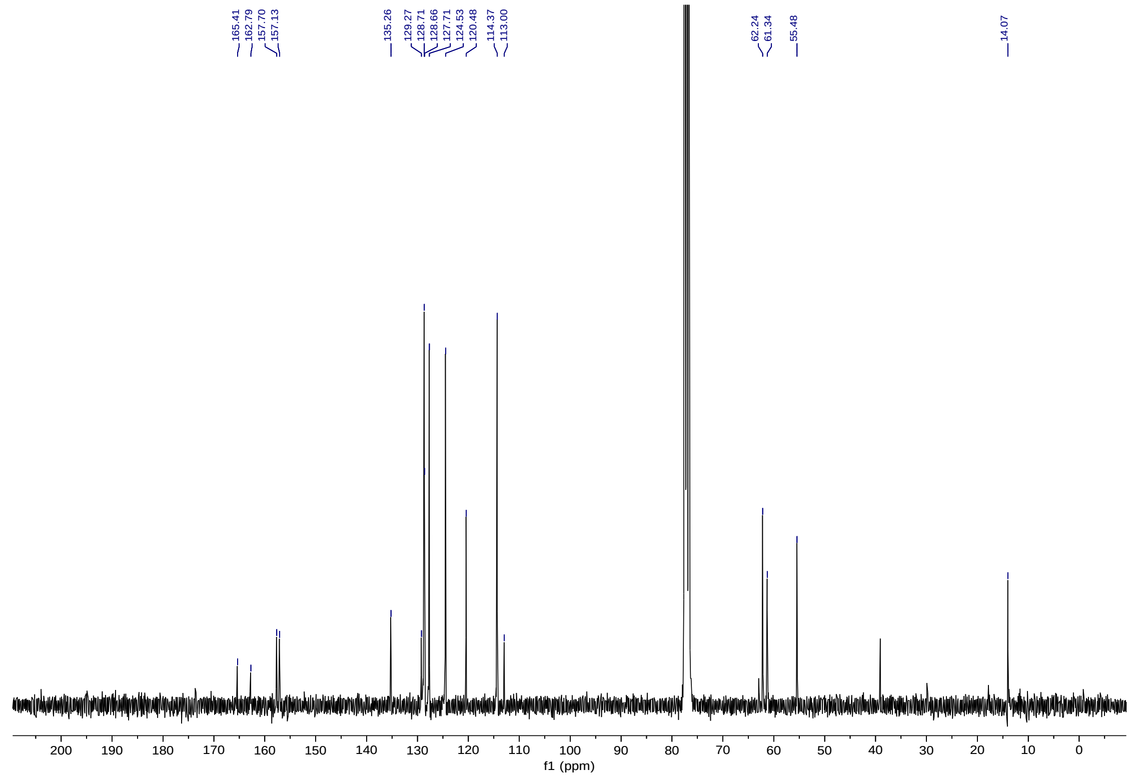

Ethyl 1-benzyl-4-hydroxy-5-oxo-2-phenyl-2,5-dihydro-1H-pyrrole-3-carboxylate. (11c).

^{1}H NMR (300 MHz, CDCl$_3$)

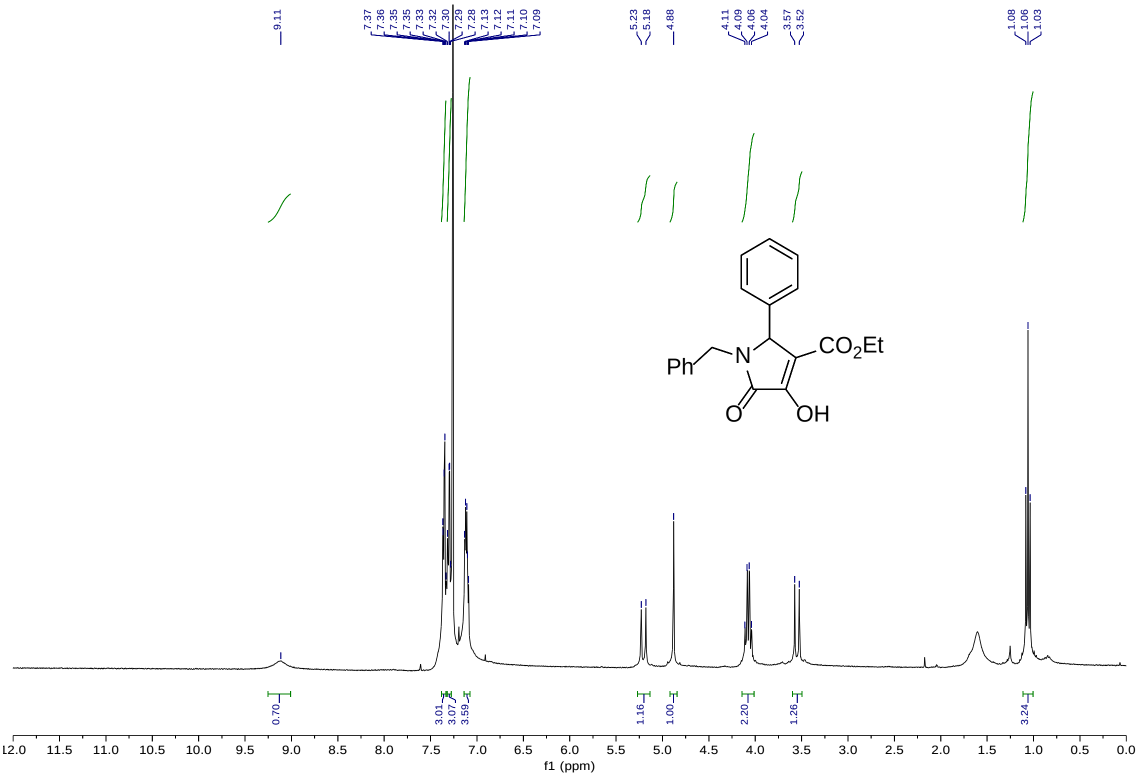

^{13}C NMR (75 MHz, CDCl$_3$)

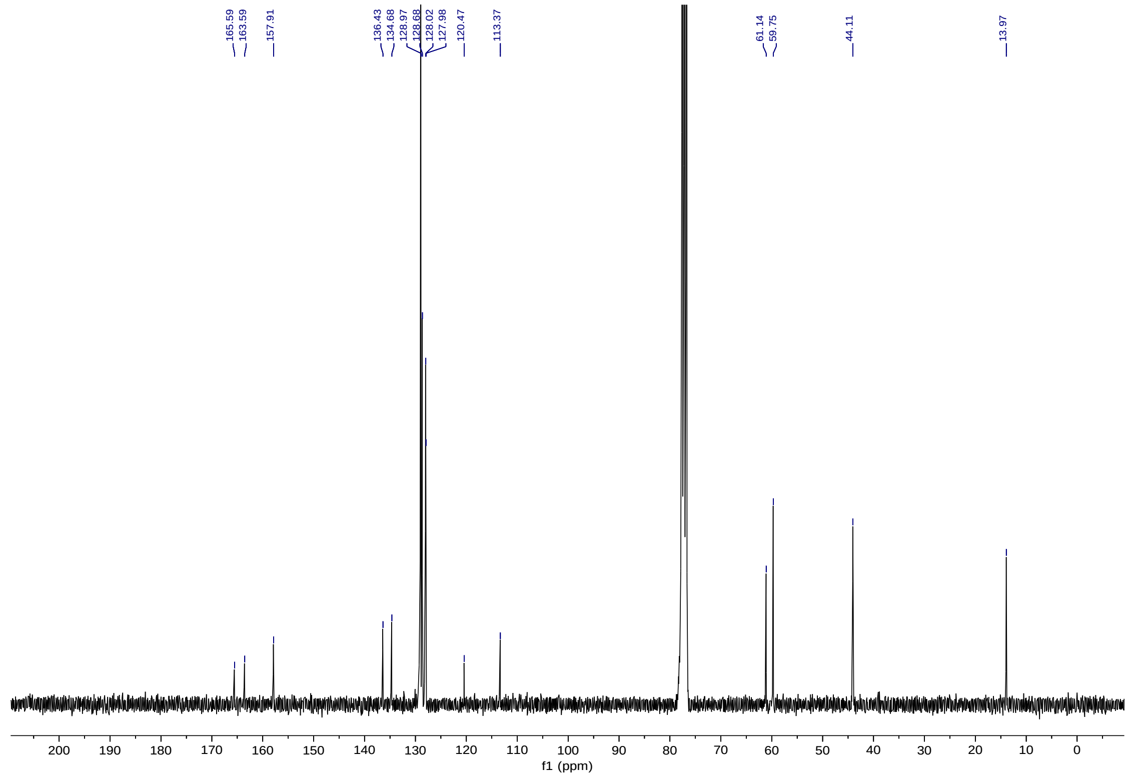

5-oxo-2-phenyl-N,1-di-p-tolyl-4-(p-tolylamino)-2,5-dihydro-1H-pyrrole-3-carboxamide (12a).

^{1}H NMR (300 MHz, CDCl₃)

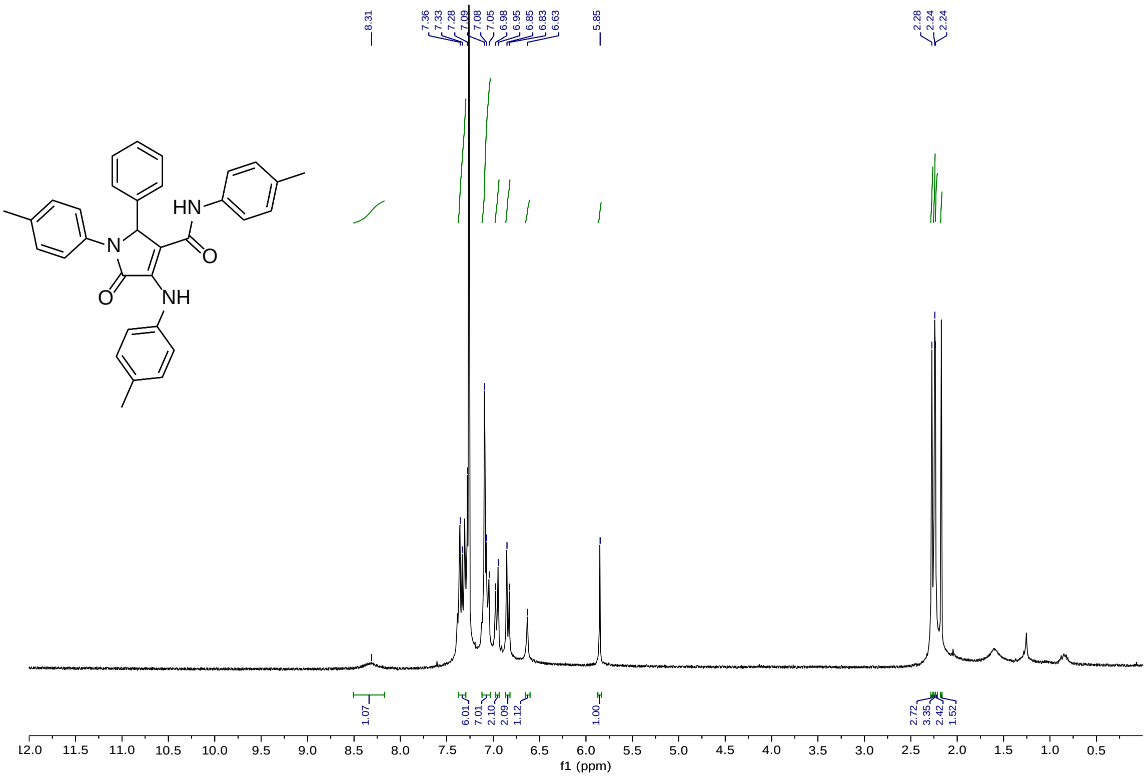

^{13}C NMR (75 MHz, CDCl₃)

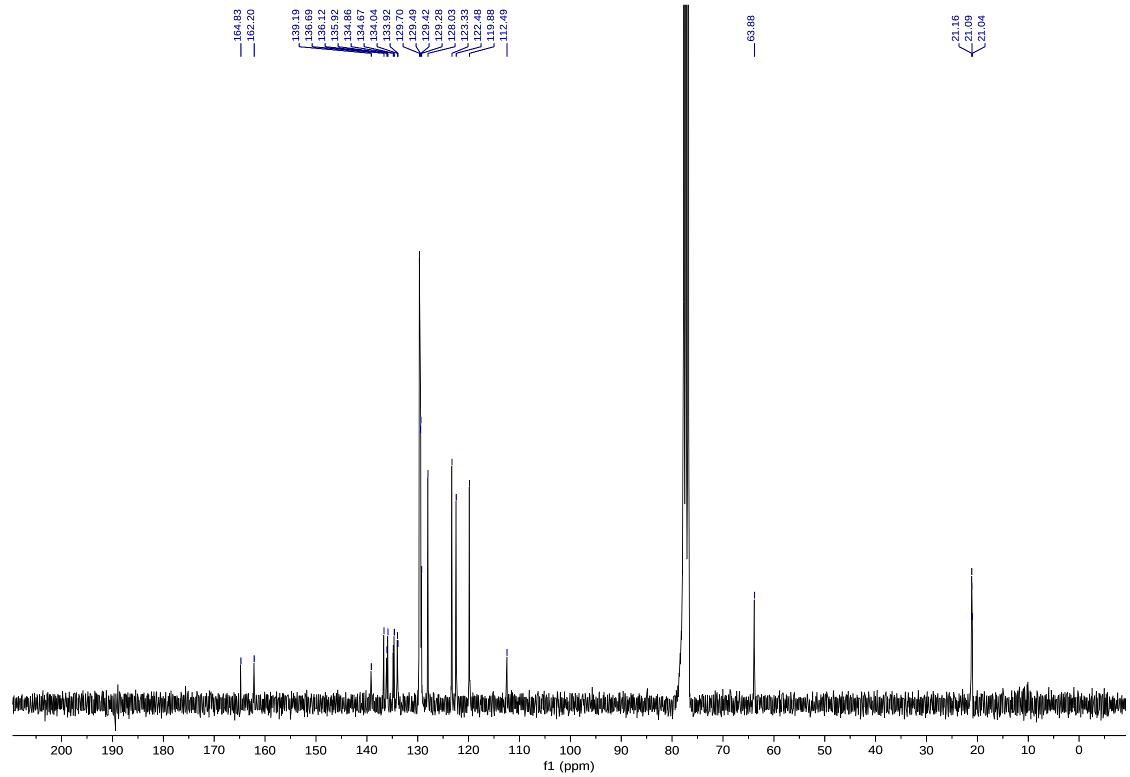

N,1-bis(4-methoxyphenyl)-4-((4-methoxyphenyl)amino)-5-oxo-2-phenyl-2,5-dihydro-1H-pyrrole-3-carboxamide. (12b).

^{1}H NMR (400 MHz, $CDCl_3$)

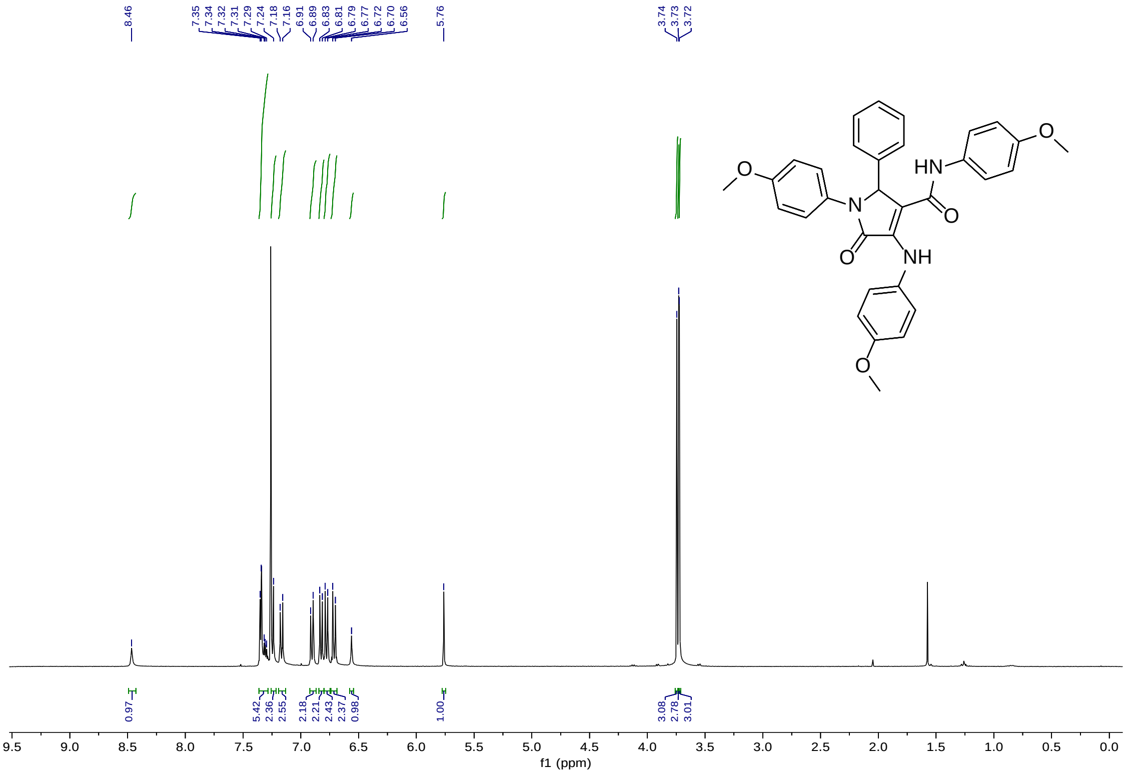

^{13}C NMR (101 MHz, $CDCl_3$)

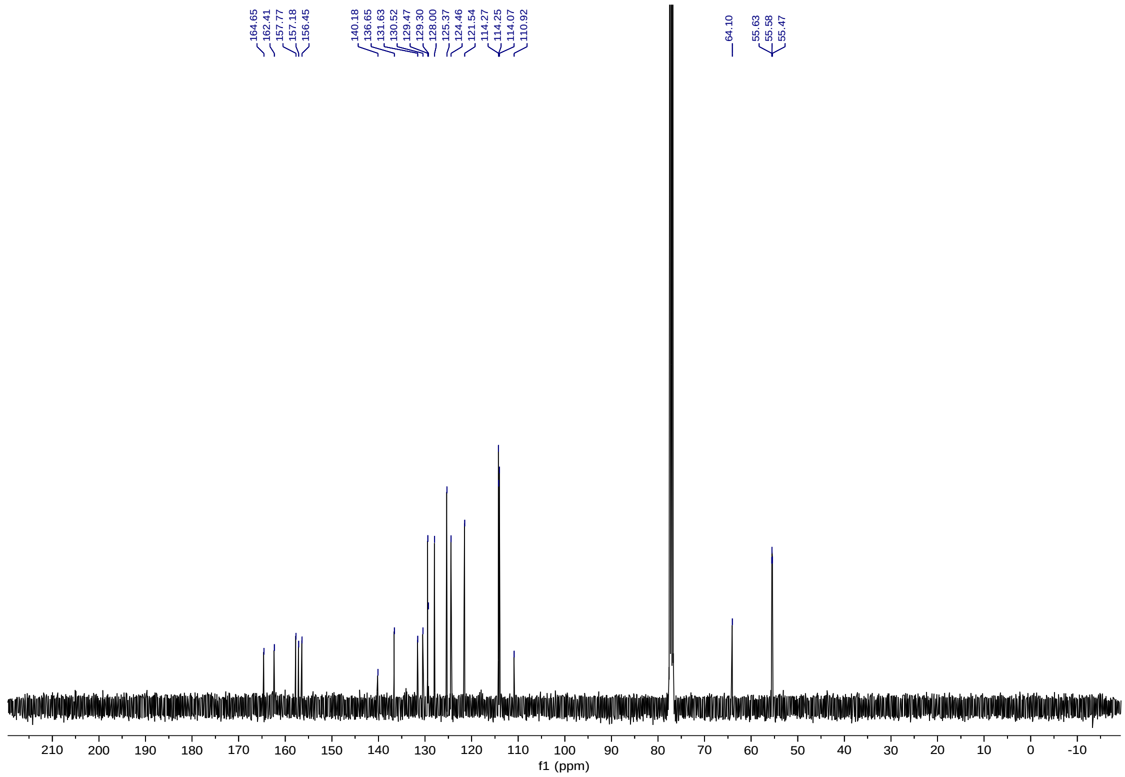

Ethyl 4-amino-1-benzyl-5-oxo-2-phenyl-2,5-dihydro-1H-pyrrole-3-carboxylate (16).

^{1}H NMR (400 MHz, CDCl$_3$)

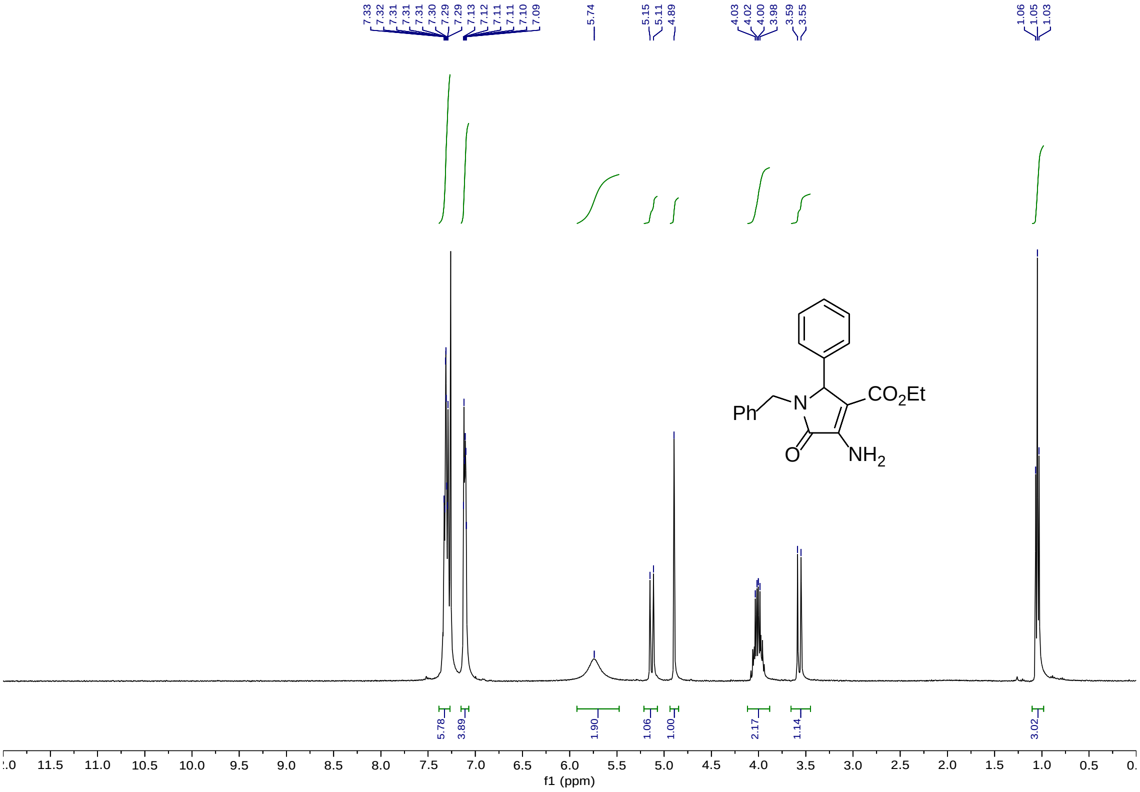

^{13}C NMR (75 MHz, CDCl$_3$)

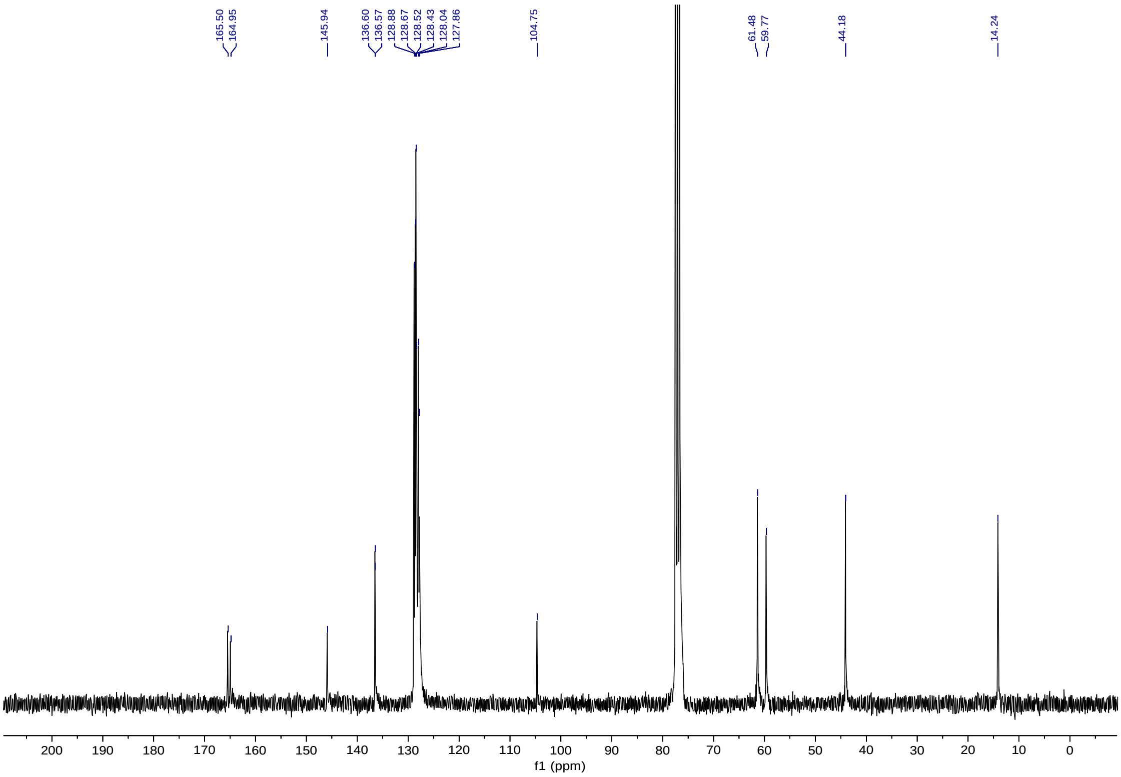

2. Crystal structure determination for compound 11a.

Thermal ellipsoid plot/ORTEP for compound 11a (50% contour probability level).

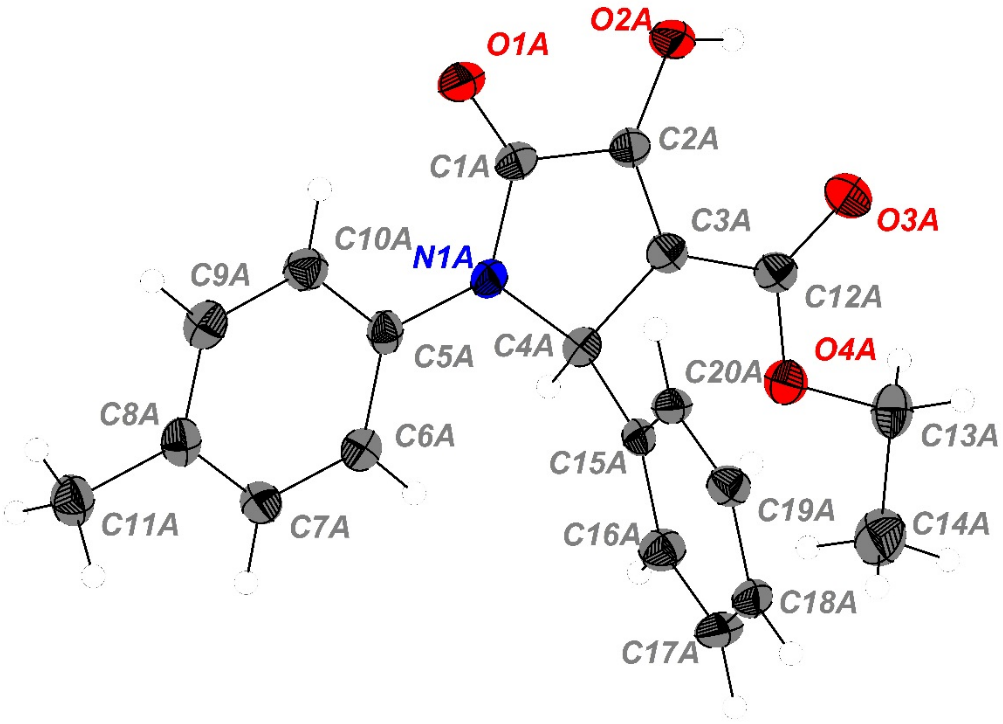

Table. Crystal data and structure refinement for 11a.

Identification code	a20180048_EM336OH
Empirical formula	$C_{20}H_{19}NO_4$
Formula weight	3376.36
Temperature/K	149.99(10)
Crystal system	triclinic
Space group	P1
a/Å	9.8131(3)
b/Å	16.1669(7)
c/Å	17.7028(7)
α/°	114.324(4)
β/°	90.139(3)
γ/°	100.214(3)
Volume/Å^3	2509.70(17)
Z	6
ρ_{calc}g/cm^3	1.339
μ/mm^{-1}	0.765
F(000)	1068.0
Crystal size/mm^3	0.257 × 0.12 × 0.052
Radiation	CuKα (λ = 1.54184)
2Θ range for data collection/°	9.19 to 137.994
Index ranges	$-9 \leq h \leq 11, -19 \leq k \leq 19, -21 \leq l \leq 21$
Reflections collected	18227

Independent reflections	9284 [R_{int} = 0.0487, R_{sigma} = 0.0647]
Data/restraints/parameters	9284/0/685
Goodness-of-fit on F^2	1.039
Final R indexes [I>=2σ (I)]	R_1 = 0.0447, wR_2 = 0.1034
Final R indexes [all data]	R_1 = 0.0662, wR_2 = 0.1148
Largest diff. peak/hole / e $Å^{-3}$	0.45/-0.21
